# Radiomic Feature Characteristics of Ovine Pulmonary Adenocarcinoma

**DOI:** 10.3390/vetsci12050400

**Published:** 2025-04-23

**Authors:** David Collie, Ziyuan Chang, James Meehan, Steven H. Wright, Chris Cousens, Jo Moore, Helen Todd, Jennifer Savage, Helen Brown, Calum D. Gray, Tom J. MacGillivray, David J. Griffiths, Chad E. Eckert, Nicole Storer, Mark Gray

**Affiliations:** 1 The Roslin Institute and Royal (Dick) School of Veterinary Studies, University of Edinburgh, Easter Bush, Edinburgh EH25 9RG, UK; 2 Moredun Research Institute, Pentlands Science Park, Bush Loan, Penicuik EH26 0PZ, UK; 3 Edinburgh Imaging Facility, Queen’s Medical Research Institute, University of Edinburgh, 47 Little France Crescent, Edinburgh EH16 4TJ, UK; 4 Centre for Clinical Brain Sciences, College of Medicine and Veterinary Medicine, University of Edinburgh, Edinburgh EH16 4SB, UK; 5 Interventional Oncology, Johnson & Johnson Enterprise Innovation, Inc., One Johnson & Johnson Plaza, New Brunswick, NJ 08933, USA

**Keywords:** ovine pulmonary adenocarcinoma, computed tomography, lung, radiomic features, sheep

## Abstract

Radiomic feature (RF) analysis of computed tomography (CT) images may aid the diagnosis and staging of ovine pulmonary adenocarcinoma (OPA). We assessed the RF characteristics of OPA tumours in JSRV-infected sheep compared to non-tumour lung tissues, examined their stability over time, and analysed RF variations in the nascent tumour field (NTF) and nascent tumour margin field (NTmF). In monthly CT scans, lung tissues were automatically segmented by density, and lung tumours were manually segmented. RFs were calculated for each imaging session, selected according to stability and reproducibility, and adjusted for volume dependence where appropriate. Comparisons between scans within sheep were facilitated through fiducial registration and spatial transformations. Initially, 9/36 RFs differed significantly from non-tumour lung tissue of similar density. Predominant RF changes included ngtdm_Complexity, glrlm_RunLNUnif_VN, and gldm_SmDHGLE. RFs in lung tumour segments showed time-dependent changes, whereas non-tumour lung tissue of similar density remained consistent. OPA lung tumour RF characteristics are distinct from those of other lung tissues of similar density and evolve as the tumour develops. Such characteristics suggest that radiomic analysis offers potential for the early detection and management of JSRV-related lung tumours. This research enhances the understanding of OPA imaging, potentially informing better diagnosis and control measures for naturally occurring infections.

## Simple Summary

Ovine pulmonary adenocarcinoma is an infectious and fatal lung cancer affecting sheep, caused by Jaagsiekte sheep retrovirus (JSRV). It is a serious economic and animal welfare issue in almost all sheep-producing countries throughout the world. As there is no vaccine or treatment available, disease control relies on identifying and culling affected sheep from flocks. Computed tomography (CT) imaging provides the current standard for delineating lung cancer pathology. When combined with radiomic feature analysis, which extracts a large number of quantitative features from medical images, the potential exists to improve clinical decision-making processes by providing detailed insights that go beyond what the human eye can perceive. In this paper, we show the potential use of radiomic analysis of sheep lung CT images for the early detection and management of JSRV-related lung tumours. This research enhances the understanding of OPA imaging, potentially informing better diagnosis and control measures for naturally occurring infections.

## 1. Introduction

Over the past ten years, radiomic feature (RF) analysis of computed tomography (CT) images has emerged as a means to extract information that objectively quantifies the spatial relationships between voxel intensities. In contrast to first-order statistics, which offer insights into the overall distribution of image intensities, second-order statistics define the relationship between neighbouring voxels, and higher-order statistics define the relationships between ‘runs’ of voxels of the same or similar intensity. Whilst the same values for first-order statistics can be derived from images of quite different visual appearance, second- and higher-order statistics vary according to appearance, particularly the ‘texture’ of the image. The large number of derived feature statistics that can be determined from CT scan data places this emerging field in the ‘-omics’ category of data science, and the biological meaning of such variation is the subject of continued exploration. Indeed, the possibility that such data might yield information on underlying tissue biology carries great promise in that imaging may yet offer a non-invasive means to characterise tissue biology and pathobiology [1,2].

In relation to lung cancer, radiomics holds great promise as CT is a long-established clinical mainstay for diagnosing and staging lung cancer, and extending the value of such images is a logical progression [3,4,5]. Cavouras et al. (1992) evaluated texture and conducted CT density matrix analysis as a means of classifying pulmonary nodules and arrived at a classification accuracy of ~90% when distinguishing benign from malignant tumours [6]. Furthermore, McNitt-Gray et al. (1999) determined that nodule classification using four textural features yielded an under the ROC curve value of 0.92 [7]. Similar findings have been reported more recently [8,9].

In the context of evaluating lung cancer in animals, the potential impact of RF analysis has yet to be fully realised. In the aim of achieving this goal, Able et al. (2021) correlated first-order RFs from canine pulmonary tumours to histopathologic characteristics and outcomes and determined that certain features were significantly associated with the latter [10]. Whilst other groups have investigated the application and potential value of machine learning for classifying abnormal chest radiographs in companion animals [11,12], a more recent review of radiomics and artificial intelligence in veterinary diagnostic imaging by Bouhali et al. (2022) highlighted the extent to which the application of such methodology lags behind human medicine [13].

Our interest in the potential of RF analysis for lung cancer relates to our research on understanding the pathogenesis of ovine pulmonary adenocarcinoma (OPA), an infectious, neoplastic lung disease of sheep that is endemic throughout most sheep-rearing countries in the world [14]. Our model, which involves experimentally infecting sheep with Jaagsiekte sheep retrovirus (JSRV), relies heavily on CT imaging to diagnose and stage lung tumour development [15]. Whilst expert radiological interpretation is a valuable adjunct to these studies, it is nonetheless in our experience that difficulties can arise in differentiating tumour tissue from other similar lung densities, particularly in the early stages of disease.

In the same way that RF analysis has been of benefit for characterising and monitoring human lung cancer [16,17], we predict that similar animal health benefits will be realised in due course. Towards this end, we undertook the following study to evaluate the RF characteristics of OPA lung tumours as an initial step towards developing systems that can aid in the diagnosis and staging of this disease. We hypothesised that the radiomic texture features of OPA lung tumours are distinct from other lung features of similar density. We further hypothesised that texture features would evolve as tumours aged.

## 2. Materials and Methods

### 2.1. Animals

This study characterises the RF changes associated with developing OPA lung lesions in twelve lambs as a consequence of the endobronchial administration of JSRV. The details of the experimental design were previously published [15], and CT imaging data related to five of the sheep from that study (sheep 14, 17, 18, 20, 24) are used herein. Imaging data from a further seven sheep which developed advanced OPA lung lesions following endobronchial infection with JSRV complete the study cohort.

Briefly, 12 lambs of mixed sex (5 female and 7 male neutered; Table 1) were commercially sourced and housed on straw bedding under standard management conditions appropriate for a research setting. Following baseline CT image acquisition, sheep were infected with JSRV via bronchoscopic instillation. CT imaging data were collected at monthly intervals until the sheep developed imaging changes consistent with OPA development. At a subsequent point dictated by overarching considerations relating to the purpose to which this model was directed, sheep were then euthanised for post-mortem examination. All imaging procedures were conducted under general anaesthesia which was conducted by veterinary specialist anaesthetists. Pre-anaesthetic medication, analgesics, induction and the maintenance of anaesthesia were performed as previously reported [15].

### 2.2. CT Acquisition and Analysis

A multi-slice SOMATOM Definition AS 64 slice fan beam CT machine was used to obtain thoracic CT scans (Siemens Healthcare Ltd., Erlangen, Germany) from each prone positioned sheep as previously described [15]. CT scans were acquired using the following parameters: 120 kVp, 73 mA, 1.2 pitch, 76.59 mm/s table speed, 23 mm/rotation table feed, 512 × 512 matrix size, and 2 mm slice thickness. Reconstructions were performed using the Siemens convolution kernel I70f2, SAFIRE strength 2. CT calibration with a phantom was performed on a weekly basis. Baseline (immediately prior to JSRV administration) and then serial thoracic CT scans were acquired at monthly intervals to assess the nature and extent of time-dependent changes in RFs. An incremental continuous positive airway pressure (CPAP) protocol was applied to induce apnoea and standardise lung volume for CT.

### 2.3. Image Segmentation

Digital Imaging and Communications in Medicine (DICOM) images were imported for segmentation into 3D Slicer (version 4.11.2, www.slicer.org, accessed on 1 January 2021). An automatic lung region segmentation module (Lung CT GMM Segmentation) developed to facilitate the quantitative analysis of CT scans from patients with coronavirus disease 19 (COVID-19) [18] was applied to segment volumes and classify sheep lung tissues according to density. This module is available as an extension for 3D Slicer at GitHub—pzaffino/SlicerDensityLungSegmentation (accessed on 16 August 2024). Averaged lung tissue segments were classified as airway (AIR), healthy lung (HL), ground glass opacities (GGO), consolidated lung (CONS), or other dense tissues (ODT).

As previously described, parenchymal tissue judged to be abnormal and consistent with an OPA lesion was separately segmented using Analyze (version 12.0 software, AnalyzeDirect, Overland Park, KS, USA) and the tumour segments imported into 3D Slicer [15]. Any potential overlap between tumour segments and other lung tissue segments (HL, GGO, CONS, and ODT) was accounted for by subtracting the former from the latter.

**Figure 1:**
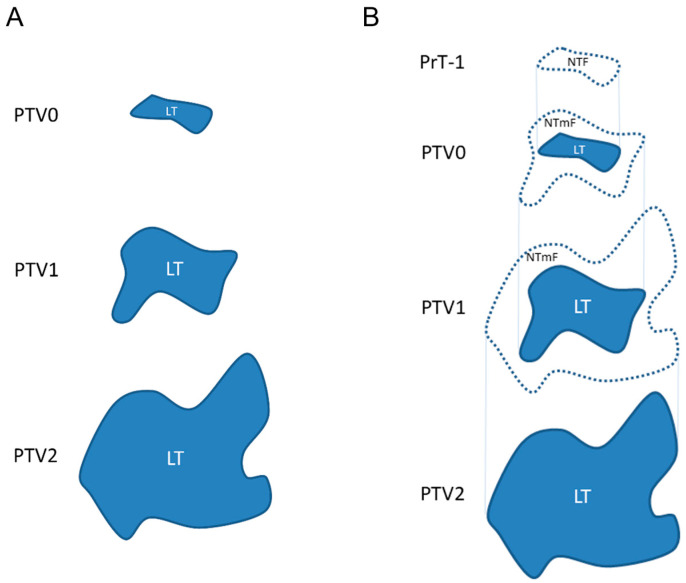
System of nomenclature for segmentation nodes. (**A**) The earliest segmentation containing a recognised lung tumour (LT) segment was labelled post-tumour volume (PTV) zero (PTV0). Subsequent segmentation nodes containing the same tumour at monthly intervals were labelled PTV1/2/3, etc. (**B**) At one month prior to PTV0 segmentation, the segmentation node was labelled pre-tumour-1 month (PrT-1). Within PrT-1, the volume where the LT would subsequently become visible was considered the nascent tumour field (NTF) segment. Similarly, after the LT formed, the surrounding volume where the tumour would become visibly evident over the course of the next month, the tumour margin field (TmF), was also considered nascent, hence the NTmF segment.

A system of nomenclature was developed to describe segmentation nodes in relation to the age of the lung tumour (LT) segment contained therein (Figure 1). The earliest segmentation containing a recognised LT segment was labelled post-tumour volume (PTV) zero (PTV0). Subsequent segmentation nodes containing the same tumour at monthly intervals were labelled PTV1/2/3, etc. The details of the CT image acquisitions relative to infection (week 0) and the associated segmentation node labels for each sheep are given in Table 2.

### 2.4. Image Processing

We previously described the assessment of feature reproducibility and stability [19] and identified 12 features that proved poorly reproducible on CT retake. These features were removed from this data set. In order to facilitate steps for reducing the dimensionality of the data set, blocks of highly correlated variables (correlation coefficient > 0.95) were identified, and redundant features were removed from each block to leave only one feature with the highest variance. The R corrplot package was used for the correlation analysis [20].

Segment volume may have a confounding effect on radiomic features [21]. In order to identify RFs demonstrating a significant volume dependence, the Spearman rank correlation between RF measurements and segment volumes was calculated. Where the association was significant (*p* < 0.05) and the absolute value of the correlation coefficient exceeded 0.4, the RF was normalised. For negative linear relationships, the RF was multiplied by segment volume, and for positive linear relationships, the RF was divided by segment volume. Nonlinear correlated RFs were corrected according to the best fit model. Following the removal of redundant features and those demonstrating poor reproducibility and stability, 36 features remained (Table 3).

### 2.5. Image Registration

In order to facilitate comparisons between sequential DICOM volumes from the same animal, fiducial markups were created using the Markups module at defined points (bifurcations) on airway trees. Through the use of the fiducial registration module, spatial transforms were created and applied to relevant LT segments, and the hardened transformed LT segments were thereafter copied to intersect with the lung segment of the preceding segmentation node. In this manner, the volumes corresponding to anticipated tumour growth could be identified.

At one month prior to the PTV0 segmentation, the segmentation node was labelled pre-tumour-1 month (PrT-1). Within PrT-1, the volume where the LT would subsequently become visible was considered the nascent tumour field (NTF) segment. Similarly, after the LT formed, the surrounding volume where the tumour would become visibly evident over the course of the next month, the tumour margin field (TmF), was also considered nascent, hence the NTmF segment (Figure 1).

The radiomics module was applied to measure the RFs of lung segments, selecting all features (resampled voxel size = 2, 2, 2; Bin Width = 64; enforced symmetrical GLCM) and saving the results to a tab-separated values (.tsv) file. The contents of each file were subsequently collated to an Excel (.xlsx) file.

### 2.6. OPA Definitive Diagnosis

OPA diagnosis was conducted as previously described [15]. Briefly, following confirmation that gross lung lesions were consistent with a diagnosis of OPA, lungs were sampled according to standardised protocols. Tissue sections were cut and stained with haematoxylin and eosin (H&E) and also subject to immunohistochemistry using an antibody raised against JSRV envelope SU to label OPA tumour cells. A board-certified veterinary pathologist examined the slides and reported on the histopathology. Cases were diagnosed as OPA-positive based on the identification of JSRV-positive cells on immunohistochemistry (IHC). No lung pathologies other than OPA were identified in these sheep.

### 2.7. Data Handling, Visualisation, and Statistics

For each segmentation node, RFs were calculated for AIR, CONS, GGO, HL, ODT, NTF, LT, and NTmF segments which, following data processing, led to the creation of a data set of 416 observations of 36 variables. A dot plot graph of the average density (Hounsfield units) of each tissue segment was created in Minitab version 22.1.0. The cluster variables function in Minitab was used to group together variables demonstrating similar patterns of expression, and these groupings were used to create heatmaps and line charts.

The statistical appraisal of time-dependent changes in RFs for each segment was conducted through a repeated measures approach, namely fitting a mixed effects model with sheep identity included as a random factor and time (BL-PTV3) as the fixed factor. The comparison of RFs associated with different lung segments, within the same animal at the same point in time, was achieved through the implementation of paired *t*-tests (*p* < 0.05). The *p*-value was adjusted for multiple comparisons using a Bonferroni correction.

## 3. Results

### 3.1. Density-Based Segmentation of Sheep Lung CT Images

**Figure 2:**
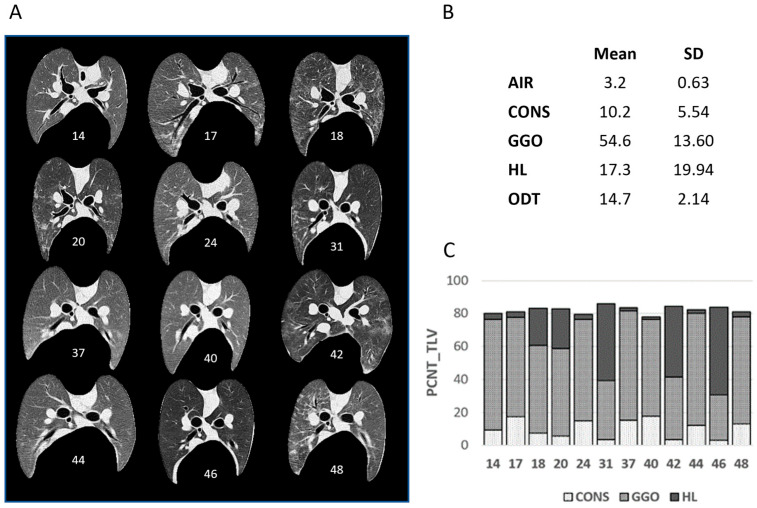
Relative proportions of lung tissue segments at baseline. (**A**) Pictogram demonstrating variability in lung density of CT images captured at baseline. (**B**) Table providing summary statistics (mean ± SD) for segment volumes, expressed as a percentage of the total lung volume (PCNT_TLV). (**C**) Bar chart highlighting inter-sheep variability in proportions of HL, GGO, and CONS segments.

The visual appraisal of sheep lung CT images at baseline indicated between-animal variability in density (Figure 2). Whilst the overall proportions of AIR and ODT segments, when considered as a percentage of the total lung volume (TLV), were relatively consistent between sheep (3.2 ± 0.63% and 14.7 ± 2.14%, respectively), the proportions of CONS (range: 3.1% to 17.6% of TLV), GGO (range: 27.6 to 68.2% of TLV), and HL (range: 1.3% to 53.1% of TLV) were more variable.

**Figure 3:**
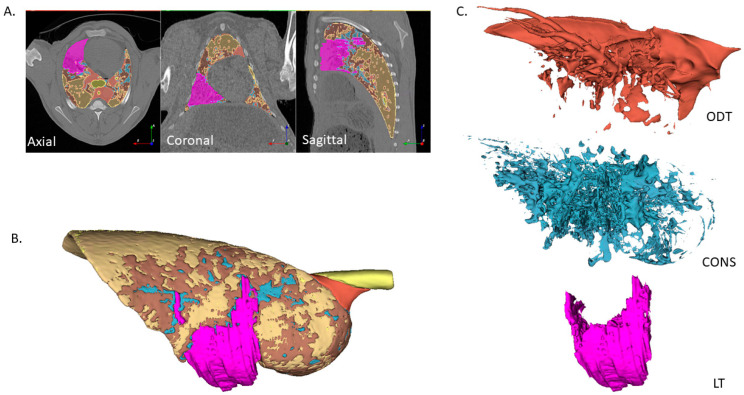
LT, CONS, and ODT segmentations. The Lung CT GMM segmentation module enabled the automatic segmentation of sheep lung CT image volumes according to density. The segmentation of LT was conducted separately using Analyze 12.0 software, and these segments were subsequently imported into the relevant averaged lung density segmentation node. LT were subtracted from other segments so that no overlap existed. Representative results of this process are depicted here (sheep 17, PTV2). (**A**) Axial, coronal, and sagittal plane CT images with the HL ■, GGO ■, CONS ■, ODT ■, and LT ■ segments indicated. (**B**) A 3-dimensional view of the right lung. (**C**) Separated CONS, ODT, and LT segments used to define lung volumes for RF analysis.

A representative example of the segmentation capabilities of the Lung CT GMM Segmentation module is depicted in Figure 3. A separately segmented tumour segment is also included. This process of segmentation facilitated the comparison of RFs between these segments.

The appraisal of CT images is influenced by the degree of lung tissue radiodensity, and different lung pathologies can share similar densities. As our interest is in determining whether RFs can be used to distinguish OPA LTs from other similar lung densities, we first sought to compare densities for the computed lung segments.

**Figure 4:**
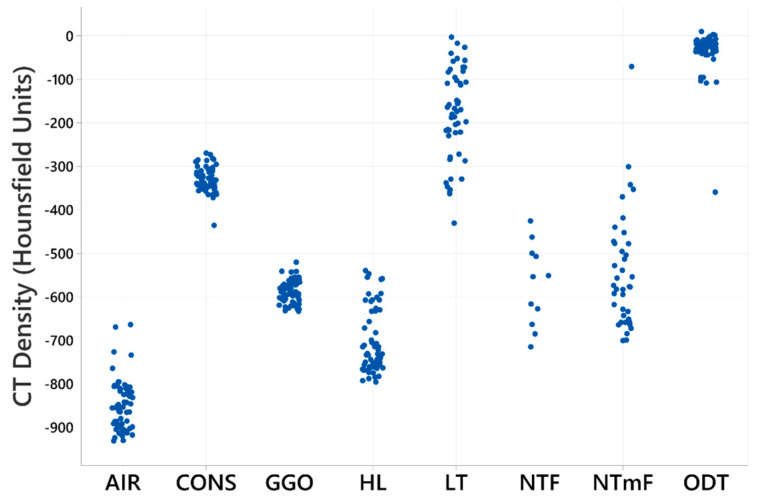
Tissue density comparisons for AIR/CONS/GGO/HL/LT/NTF/NTmF and ODT segments. Dot plot depicting average tissue density (in Hounsfield units) for lung tissue volumes defined by AIR/CONS/GGO/HL/LT and ODT segments. Data indicate that densities of lung tissue defined by CONS, ODT, and LT segments show overlap and are distinct from lung tissue densities defined by AIR, HL, and GGO segments.

Measured densities for the CONS, ODT, and LT segments overlapped and were distinct from those of GGO and HL (Figure 4). This observation directed a further analysis towards determining the RF characteristics of OPA tumours through comparing these segments.

### 3.2. Radiomic Feature Characteristics of LT Segments

**Figure 5:**
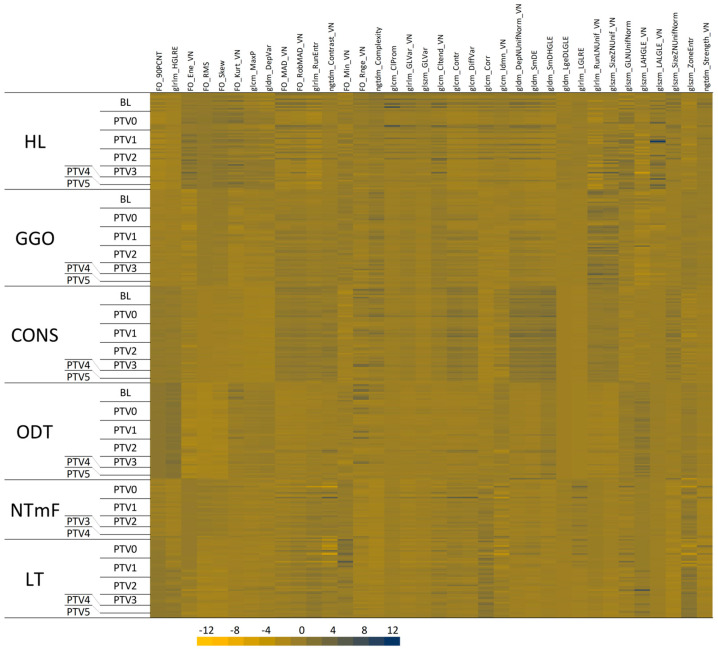
Heatmap representation of radiomic features. CT images containing lung tumours (LT) were identified, and segmentation nodes were created in 3D Slicer to accommodate LT segments. In addition, the nascent tumour margin field (NTmF), where a tumour would develop over the course of the next month, was identified through the process of backwards registration to superimpose the subsequent tumour volume on the pre-existing lung volume. Segments representing HL, GGO, CONS, and ODT were also identified. Segmentation nodes were labelled according to the age of the tumour, from PTV0 when the tumour was first identified, through monthly increments (PTV1, PTV2, etc.) thereafter. RFs were calculated for these segments, standardised (by subtracting the mean and dividing by the standard deviation), and clustered. The panels in this heatmap facilitate the visualisation of RFs in LT and NTmF relative to other lung tissues of similar density and the changes in these features with time.

A heatmap representation of these RFs suggests that RFs vary between the studied segments and that some LT features show time-dependent changes (Figure 5).

**Figure 6:**
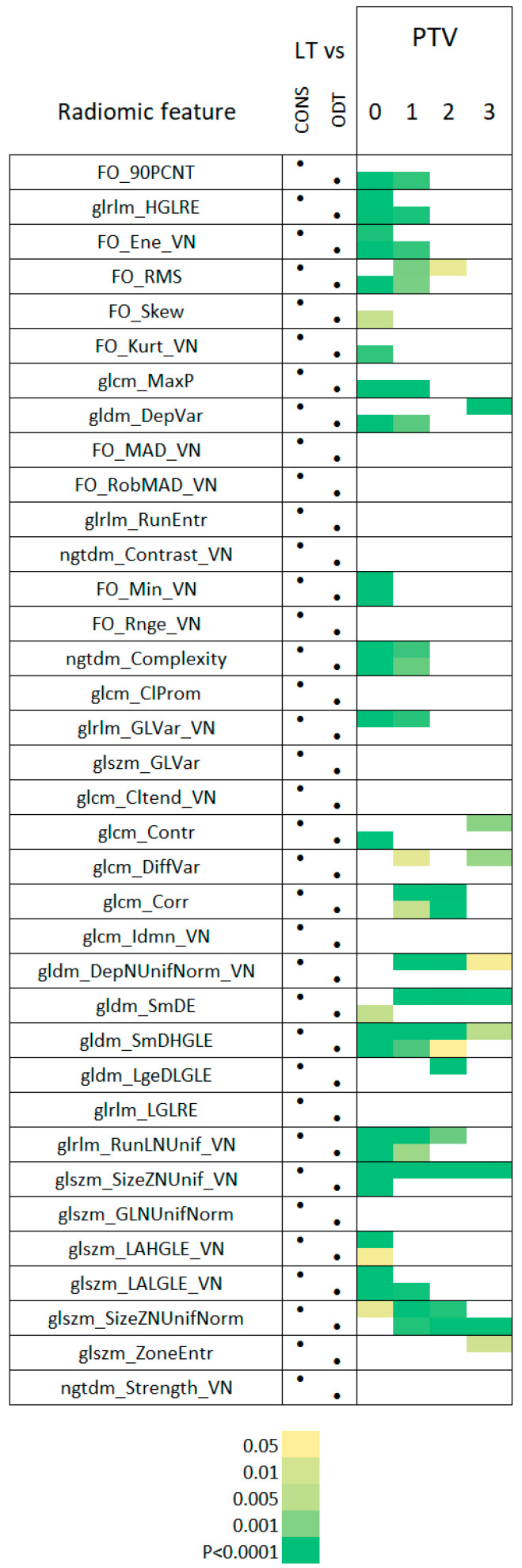
RFs of OPA tumours relative to similar lung densities. The RFs are indicated in the first column. Paired *t*-tests with Bonferroni correction for multiple comparisons were applied to assess whether any significant (*p* < 0.05) difference in RFs existed when comparing between LT and CONS and LT and ODT. This comparison was implemented for every segmentation containing a recognised lung tumour (LT) segment (PTV0-5), with the nature of the comparisons indicated in the CONS and ODT columns. The significance of comparisons can be assessed through reference to the legend. The data indicates that many RFs of OPA LT segments differ significantly from those of the CONS and ODT segments. This difference is particularly evident for early-stage LTs (PTV0), where 9/36 RFs differed significantly from those associated with both the CONS and ODT segments. Thereafter, consistent differences relative to both the CONS and ODT segments were rarely noted—with the exceptions of ngtdm_Complexity, glrlm_RunLNUnif_VN (PTV1), and gldm_SmDHGLE (PTV1 and 2).

**Figure 7:**
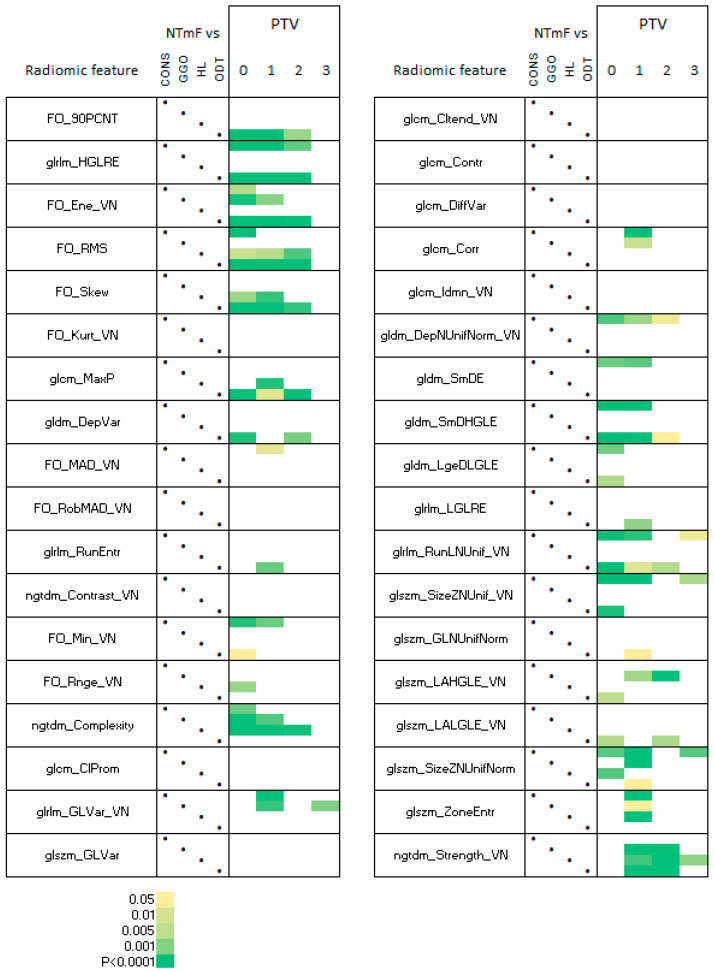
RFs of NTmF relative to other lung densities. The significance (paired *t*-tests with Bonferroni correction for multiple comparisons) of paired comparisons between NTmF and other lung segments defined on the basis of image density (HL, GGO, CONS, and ODT) is depicted. The nature of the comparisons is indicated in the CONS, GGO, HL, and ODT columns. The density of NTmF segments predominantly overlaps with the HL and GGO segments; hence, there is particular relevance attached to these comparisons. ngtdm_Complexity was found to differ significantly from the GGO and HL segments at PTV0 and 1, and ngtdm_Strength_VN differed significantly from that of these segments at PTV1 and 2.

Paired *t*-tests with Bonferroni correction for multiple comparisons were applied to assess whether any significant (*p* < 0.05) difference in RFs existed when comparing between LT and CONS, and LT with ODT segments (Figure 6), and between NTmF and HL, GGO, CONS, and ODT segments (Figure 7). The number of sheep decreased during the course of this study (Table 2); hence, statistical power was gradually lost. Twelve paired comparisons were evaluated for PTV0 and PTV1, 11 for PTV2, 7 for PTV3, and 5 for PTV4, with only 3 available for PTV5. Only one sheep had data available for PTV6 segmentation.

Recognising that the decline in statistical power would potentially undermine statistical inference, only the initial time points (PTV0-PTV3) were included in the paired analysis, these results are depicted in Figure 6 and Figure 7.

**Figure 8:**
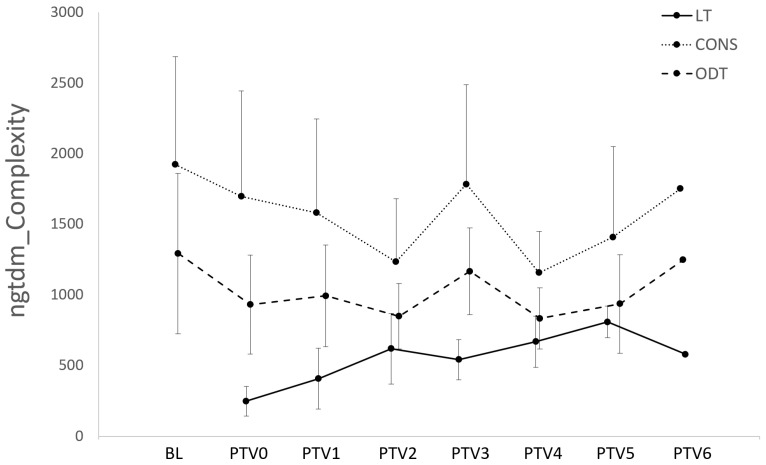
Time-dependent changes in ngtdm_Complexity for LT, CONS, and ODT segments. Line chart demonstrating relative levels of ngtdm_Complexity for LT, CONS, and ODT segments over time. Levels of this RF in LT segments differed significantly from those of both CONS and ODT segments in PTV0 and PTV1 segmentation nodes (*p* < 0.05, Figure 6). Error bars represent standard deviation. Initial cohort (*n* = 12) declined in size during period of observation as follows: PTV0 and PTV1 *n* = 12; PTV2 *n* = 11; PTV3 *n* = 7; PTV4 *n* = 5; PTV5 *n* = 3; and PTV6 *n* = 1.

Many RFs of OPA LT segments differed significantly from those of the CONS and ODT segments. This difference was particularly evident for early-stage LTs (PTV0), where 9/36 RFs differed significantly from those associated with both the CONS and ODT segments. Thereafter, consistent differences relative to both the CONS and ODT segments were more rarely noted—with the exceptions of ngtdm_Complexity, glrlm_RunLNUnif_VN (PTV1), and gldm_SmDHGLE (PTV1 and 2). The respective levels of ngtdm_Complexity in the LT, CONS, and ODT segments are depicted in Figure 8.

**Figure 9:**
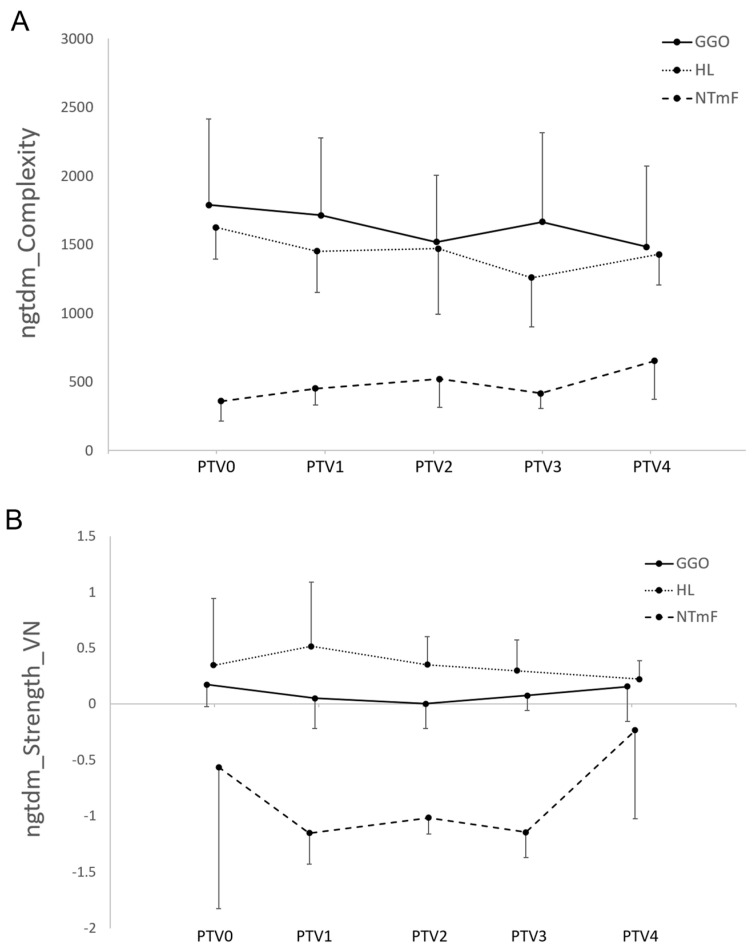
Line charts demonstrating relative levels of (**A**) ngtdm_Complexity and (**B**) ngtdm_Strength_VN for NTmF, HL, and GGO segments over time. Levels of ngtdm_Complexity in NTmF segments differed significantly from both HL and GGO segments at PTV0 and 1, and ngtdm_Strength_VN differed significantly from these segments at PTV1 and 2 (*p* < 0.05, Figure 7). Error bars represent standard deviation. Initial cohort (*n* = 12) declined in size during period of observation as follows: PTV0 and PTV1 *n* = 12; PTV2 *n* = 11; PTV3 *n* = 7; PTV4 *n* = 5; PTV5 *n* = 3; and PTV6 *n* = 1.

In relation to the NTmF, the levels of ngtdm_Complexity in NTmF segments differed significantly from those of both the HL and GGO segments at PTV0 and 1, and ngtdm_Strength_VN differed significantly from that of these segments at PTV1 and 2 (Figure 9).

### 3.3. Radiomic Features of LT Segments Demonstrate Time-Dependent Changes

Repeated measures analysis confirmed that a number of features changed significantly between BL and PTV3. Table 4 shows these RFs, as well as indicating whether the change represented an increase or decrease over time. LT demonstrated significant directional changes in 9/36 of the RFs, whereas a combined total of only 5 features from the other segments demonstrated significant directional changes.

### 3.4. Radiomic Features of NTF Segments

**Figure 10:**
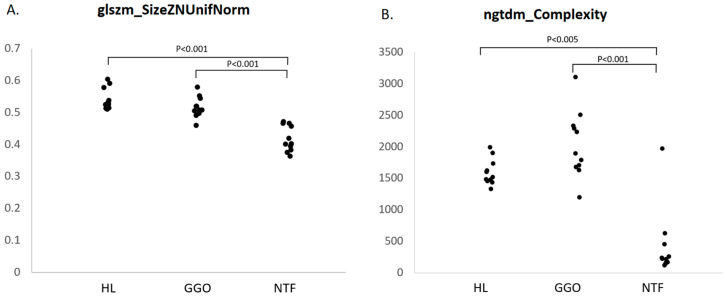
Comparison of glszm_SizeZNUnifNorm and ngtdm_Complexity in NTF, HL, and GGO segments. Dot plots representing values of (**A**) glszm_SizeZNUnifNorm and (**B**) ngtdm_Complexity for NTF, HL, and GGO segments one month prior to the appearance of LT. Paired *t*-tests with correction for multiple comparisons established that the levels of these features were significantly lower in NTF segment when compared to HL and GGO.

The availability of RF data relating to the nascent tumour field (NTF) enabled a comparison with lung segments representing healthy lung and ground glass opacity, asking whether RF changes precede the development of visible lung tumours. NTF segment data were available for eleven sheep (in one sheep, RF characterisation could not be achieved due to the small size of the NTF segment). Paired *t*-tests with correction for multiple comparisons established that the levels of the measured features glszm_SizeZNUnifNorm and ngtdm_Complexity were significantly lower in the NTF segment when compared to HL and GGO (Figure 10).

A schematic overview of the experimental approach is provided in the Supplementary Materials (Figure S1).

## 4. Discussion

Our interest in RF analysis of OPA developed from the observation that the definitive radiological diagnosis of this disease is subject to uncertainty. As radiomic analysis can furnish insights into image characteristics that lie beyond visual acuity, we reasoned that OPA LTs may demonstrate RF characteristics distinct from similar lung densities and sought to address this hypothesis. Whilst there is an implicit assumption that RF changes reflect underlying tissue biology, this was not directly assessed in this study. Nonetheless, some reflection on OPA tissue biology is pertinent.

Whilst JSRV will infect any cells expressing hyaluronoglucosaminidase 2, the particular productive tropism that JSRV has for proliferating type 2 pneumocytes relates to the fact that the viral long terminal repeat (LTR) contains enhancer elements that bind the transcription factors expressed by these cells [22]. The JSRV envelope glycoprotein (env) is itself oncogenic, directly stimulating neoplastic cell transformation and promoting cell proliferation [23]. The consequent expression, packaging, and release of the virus leads to the further dissemination of infection both within the lung and when exhaled, to potentially infect other sheep. Hence, it is the release of infectious virus that leads to new infection and transformation events initiating new foci and further tumour growth. The proliferating epithelial cells impose into the airspaces, with the tumour foci remaining devoid of any capsule but otherwise supported by a fibrovascular connective tissue scaffold and infiltrated with macrophages and lymphocytes. The centre of large tumours may become necrotic.

The imaging features of OPA may therefore reflect all aspects of this process, from the earliest histological growth of tumour acini through to large tumour masses with organised connective tissue scaffolds and central necrosis. In this study, the veracity of manual tumour segmentations was ultimately confirmed through gross and histopathological assessment at necropsy examination, providing the reassurance of prior manual segmentations.

Some variation in the time taken from infection to the first identification of a tumour on CT was noted, with most sheep developing early tumours by 8 weeks, but one sheep was delayed to the extent of 36 weeks. As sheep were group-housed, it is possible that the latter reflects a new infection event as a consequence of being exposed to virus shed from another sheep sharing the same airspace. Regardless, we adopted a strategy to evaluate sheep on the same footing, that is, in relation to the time point at which the tumour was visible on CT, rather than the point at which all sheep were exposed to infection. The basis of variable susceptibility to the consequences of JSRV infection, whether in relation to microscopic or gross lung tumour growth, has yet to be elucidated.

The utility of the automatic lung segmentation process was a fundamental benefit to this study, enabling the density-based rapid classification of sheep lung tissue segments, usually within 2–3 min on a desktop PC (ACPI x64-based PC Intel^®^ Core™ i5-9500 CPU @ 3GHz; Hewlett-Packard, Palo Alto, CA, USA). The density of manually segmented LT overlapped the CONS and ODT segments. Whilst the former is not considered a feature of healthy lungs, the latter is and is largely comprised of intrapulmonary and mediastinal blood vessels. The manual segmentation of LT enabled the subtraction of perceived tumour tissue from the CONS and ODT segments. Similarly, NTmF segment densities predominantly overlapped with the HL and GGO segments, hence directing RF comparisons with these segments.

Whilst exhibiting structural and physiological similarities with human lungs, it is important to acknowledge that the above classification applied to sheep CT images carries no assumption that the underlying tissue-level structural and functional features are equivalent between these species. Indeed, a classification of GGO in human lung CT images usually represents abnormality, whereas such classification is commonly recognised throughout the lungs of healthy anaesthetised sheep, as seen in the baseline scans of the sheep used in this study.

We established that LTs are characterised by RFs distinct from those associated with other lung tissues of similar density, and we also identified patterns of change in RF characteristics as LTs aged. The observation that LT RFs tended to demonstrate significant differences only at the earlier time points should be cautiously interpreted as statistical power was lost for this comparison as sheep were removed from this study.

Our results further demonstrate that the RF characteristics of LTs change as the tumour ages. As previously stated, the cellular and structural features associated with tumour growth do change over time; hence, this finding is not intuitively surprising. However, our previous data illustrate that tumour volume doubling times following experimental infection with JSRV [15], whilst bearing comparison with field data [24], do indicate that tumour growth rates can vary considerably between sheep and also within sheep over time. Hence, the statistical evidence of time-dependent changes in RFs is perhaps more surprising in this context.

This observation raises several possibilities that might be relevant to future research utility. The most obvious is the concept that RF analysis might be of use for staging disease and that this may hold relevance for disease in the field. Whilst CT imaging holds no practical relevance for screening endemically infected flocks on a routine basis, the possibility of staging disease at a point in time might however have advantages in the context of modelling prevalence and incidence across different age groups and/or the likely consequences of different control strategies.

It is likely that surrounding each manual tumour segmentation exists a peripheral zone that reflects the spread of the growing tumour. Whilst invisible to the eye, the underlying changes in tissue biology might nonetheless possess texture characteristics that can be captured by an RF analysis of this nascent tumour margin field. Our results confirm this to be the case, with the levels of ngtdm_Complexity and ngtdm_Strength_VN found to be significantly lower in the NTmF segment when compared to the GGO and HL segments (at PTV0 and 1 for ngtdm_Complexity and PTV1 and PTV2 for ngtdm_Strength_VN). The NGTDM quantifies the difference between a grey value and the average grey value of its neighbours within a defined distance. Complexity measures the non-uniformity of an image, specifically where there are many rapid changes in grey-level intensity. NGTDM strength values are high when the primitives in an image are easily defined and quite distinguishable. In the case of NTmF, the primitives are smaller and less distinguishable relative to the GGO and HL segments.

Our own future work will endeavour to examine the relationship between the RFs of the NTmF and underlying tissue biology. Similarly, the age characterisation of particular tumours, volumes within tumours, or suspected tumours might offer insights into disease pathogenesis when coupled with the utility of bronchoscopic and/or transthoracic tumour biopsy.

Finally, we identified the NTF through the ‘backwards’ registration of the tumour volume (LT) at the point it first became visually apparent (PTV0) to the segmentation made one month previously (PrT-1). It should be appreciated that there is considerable potential for error in this process in that the initially visualised LT was small, with 8/12 LTs being smaller than 15 cm^3^; this increases the chance that the registration process may not have afforded perfect alignment with the ‘true’ NTF. However, we did identify that the levels of glszm_SizeZNUnifNorm and ngtdm_Complexity were significantly lower in the NTF segment when compared to the HL and GGO segments. Both features were highlighted in previous comparisons, the former in relation to comparing LT against CONS and ODT and notably the latter in the context of comparisons between the NTmF and surrounding lung (GGO and HL). Hence, we are motivated by the fact that the NTF is indeed characterised by RF changes relative to the surrounding lung. However, a further possibility exists, namely that the RF changes attributed to the NTF extended to a greater degree throughout the initially infected segment but not to the extent that the values for HL and GGO in the lungs were impacted. This might also imply that microscopic tissue changes are initially more widespread than would be imagined on the basis of the first visual appreciation of LT.

Currently, ultrasound analysis is the only practical tool available to veterinarians and farmers which is capable of identifying sheep with gross lung tumours affecting the imaging-accessible portions of the lungs. This rapid and low-cost approach can be used to screen flocks and identify sheep that can be culled or managed separately in order to remove infectious burden and prevent the infection of susceptible sheep. Whilst the conventional analysis of ultrasound images is based on the identification of features that relate to the presence of gross lung tumours, the notion that changes to lung biology as a consequence of early-stage disease might be amenable to more sophisticated analysis bears consideration. Future work, in extending to the quantification of the lung ultrasound image texture characteristics of sheep experimentally infected with JSRV, will be fundamental to exploring this possibility.

OPA is often cited as a potentially useful model for human lung adenocarcinoma. Adenocarcinoma is a histological subtype of non-small cell lung carcinoma (NSCLC), the most common form of lung cancer (80–85%). Early OPA demonstrates a lepidic growth pattern, where transformed alveolar epithelial cells proliferate along the alveolar walls without invading the lung stroma. A recent human clinical study examined the potential of radiomics for identifying lung adenocarcinomas with predominant lepidic growth in pure ground glass nodules [25] and determined that the application of CT radiomics did improve identification performance. Later advanced OPA lesions more closely resemble adenocarcinoma with a papillary or acinar-predominant pattern [26]. The potential of peri-tumoural CT RFs to predict the prognosis of human lung cancer is currently being reported in the literature [27,28,29]. Other studies have established the value of combining both intra- and peri-tumoural RFs in predicting the prognosis of pure-solid non-small cell lung cancer [30] and differentiating early-stage lung invasive adenocarcinoma (≤3 cm) subtypes [31]. Notably, Wu et al. (2023) recently conducted a systematic review evaluating the value of peri-tumoral CT RFs for predicting the prognosis of non-small cell lung cancer; they concluded that studies demonstrated promise in predicting the prognosis of NSCLC and also determined that the variability and heterogeneity amongst the methodology, analysis, and reporting of the included studies warrants caution in interpretation at this stage in time [27].

Such comparisons serve to indicate the likely direction that radiomics will follow in the veterinary field and, in particular, outline the scope and extent of studies necessary for generating predictive models capable of being used on image data sourced from multiple centres.

## 5. Conclusions

Our study demonstrates that OPA lung tumours arising as a consequence of endobronchial infection with JSRV have RF characteristics distinct from other lung tissues of similar density in the same sheep, that these characteristics change as the tumour grows, and that pre-visual RF changes are associated with both the nascent tumour field and the nascent tumour margin field. This is a promising initial step towards generating models capable of diagnosing and staging OPA lung pathology, indicating the future use of such tools for understanding disease pathogenesis.

## Figures and Tables

**Table 1:** Signalment and body weights of sheep at the time of JSRV instillation. All sheep were crossbred (Texel X mule). Details pertaining to five sheep (14, 17, 18, 20, and 24) were previously reported [15]. Five female (F) and seven male neutered (MN) sheep were studied.

Sheep ID	Age (Months)	Sex	Body Weight (kg)
14	5	MN	41
17	5	F	47
18	5	F	35
20	5	F	37
24	5	MN	38
31	4	F	20
37	4	F	25
40	4	MN	22
42	4	MN	30
44	4	MN	25
46	4	MN	20
48	4	MN	30

**Table 2:** Details of the CT image acquisitions, relative to infection (week 0) and the associated segmentation node labels for each sheep. The earliest segmentation containing a recognised lung tumour (LT) segment was labelled post-tumour volume (PTV) zero (PTV0). Subsequent segmentation nodes containing the same tumour at monthly intervals were labelled PTV1/2/3, etc. Grey area indicates the sheep was euthanised and no further CT imaging was performed.

	Week										
**Sheep**	0	4	8	12	16	20	24	28	32	36	40
14			PTV0	PTV1	PTV2						
17				PTV0	PTV1	PTV2	PTV3				
18			PTV0	PTV1	PTV2	PTV3	PTV4				
20					PTV0	PTV1	PTV2	PTV3			
24			PTV0	PTV1	PTV2	PTV3	PTV4	PTV5			
31			PTV0	PTV1	PTV2	PTV3	PTV4	PTV5	PTV6		
37			PTV0	PTV1	PTV2						
40			PTV0	PTV1	PTV2						
42		PTV0	PTV1	PTV2							
44			PTV0	PTV1	PTV2	PTV3	PTV4	PTV5			
46			PTV0	PTV1	PTV2	PTV3	PTV4				
48										PTV0	PTV1

**Table 3:** Radiomic features used in the analysis. Following removal of redundant features and those features exhibiting poor reproducibility and stability, 36 features remained for use in the analysis. For purpose of clarity, RFs are abbreviated throughout this manuscript. Abbreviations are defined in this table. Features which were subject to volume normalisation have suffix ‘_VN’ added to their abbreviation.

Feature Class	Features
First Order	90th Percentile (FO_90PCNT), Energy (FO_Ene_VN), Kurtosis (FO_Kurt_VN), Mean Absolute Deviation (FO_MAD_VN), Minimum (FO_Min_VN), Root Mean Squared (FO_RMS), Range (FO_Rnge_VN), Robust Mean Absolute Deviation (FO_RobMAD_VN), Skewness (FO_Skew)
GLCM Gray Level Co-occurrence Matrix	Cluster Prominence (glcm_ClProm), Cluster Tendency (glcm_Cltend_VN), Contrast (glcm_Contr), Correlation (glcm_Corr), Difference Variance (glcm_DiffVar), Inverse Difference Moment Normalized (glcm_Idmn_VN), MaximumProbability (glcm_MaxP)
GLDM Gray Level Dependence Matrix	Dependence Non-Uniformity Normalized (gldm_DepNUnifNorm_VN), Dependence Variance (gldm_DepVar), Large Dependence Low Gray Level Emphasis (gldm_LgeDLGLE), Small Dependence Emphasis (gldm_SmDE), Small Dependence High Gray Level Emphasis (gldm_SmDHGLE)
GLRLM Gray Level Run Length Matrix	Gray Level Variance (glrlm_GLVar_VN), High Gray Level Run Emphasis (glrlm_HGLRE), Low Gray Level Run Emphasis (glrlm_LGLRE), Run Entropy (glrlm_RunEntr), Run Length Non-Uniformity (glrlm_RunLNUnif_VN)
GLSZM Gray Level Size Zone Matrix	Gray Level Non-Uniformity Normalized (glszm_GLNUnifNorm), Gray Level Variance (glszm_GLVar), Large Area High Gray Level Emphasis (glszm_LAHGLE_VN), Large Area Low Gray Level Emphasis (glszm_LALGLE_VN), Size Zone Non-Uniformity (glszm_SizeZNUnif_VN), Size Zone Non-Uniformity Normalized (glszm_SizeZNUnifNorm), Zone Entropy (glszm_ZoneEntr)
NGTDM Neighbouring Gray Tone Difference Matrix	Complexity (ngtdm_Complexity), Contrast (ngtdm_Contrast_VN), Strength (ngtdm_Strength_VN)

**Table 4:** Results of the repeated measures analysis conducted to determine whether the radiomic features of lung segments change significantly over time. A repeated measures approach was used. A mixed effects model with sheep identity included as a random factor and time (BL—PTV3) as the fixed factor determined that significant changes over time were featured predominantly for the LT segment, where a significant directional change was identified in 9/36 of the RFs. This table indicates the significance and direction of the change (increased ↑, or decreased ↓) for individual features (* *p* < 0.001 and ** *p* < 0.0001).

	AIR		HL		GGO		CONS		LT		NTmF		ODT		
glrlm_HGLRE									**	↑					
FO_Ene_VN									**	↓					
FO_Skew					**	↓	**	↓							
gldm_DepVar									**	↑					
ngtdm_Complexity			**	↓					**	↑					
glcm_Idmn_VN	*	↑													
gldm_SmDE									**	↓					
glrlm_LGLRE									*	↓					
glszm_SizeZNUnif_VN									**	↓					
glszm_LALGLE_VN									**	↑					
glszm_ZoneEntr									**	↑					
ngtdm_Strength_VN	*	↓													

## Data Availability

The original contributions presented in this study are included in the article. Further inquiries can be directed to the corresponding author.

## Supplementary Materials

The following supporting information can be downloaded at https://www.mdpi.com/article/10.3390/vetsci12050400/s1, Figure S1: Schematic overview of the experimental approach.

## Abbreviations

The following abbreviations are used in this manuscript:

BL	Baseline
ClProm	Cluster Prominence
Cltend	Cluster Tendency
CONS	Consolidated
Contr	Contrast
Corr	Correlation
CPAP	Continuous positive airway pressure
CT	Computed tomography
DepNUnifNorm	Dependence Non-Uniformity Normalized
DepVar	Dependence Variance
DICOM	Digital Imaging and Communications in Medicine
DiffVar	Difference Variance
Ene	Energy
env	JSRV envelope glycoprotein
FO	First order
GGO	Ground glass opacity
glcm	Gray-Level Co-occurrence Matrix
GLCM	Graylevel co-occurrence matrix
gldm	Gray Level Dependence Matrix
GLNUnifNorm	Gray Level Non-Uniformity Normalized
glrlm	Gray Level Run Length Matrix
glszm	Gray Level Size Zone Matrix
GLVar	Gray Level Variance
H&E	Haematoxylin and eosin
HGLRE	High Gray Level Run Emphasis
HL	Healthy lung
Idmn	Inverse Difference Moment Normalized
IHC	Immunohistochemistry
JSRV	Jaagsiekte sheep retrovirus
Kurt	Kurtosis
LAHGLE	Large Area High Gray Level Emphasis
LALGLE	Large Area Low Gray Level Emphasis
LgeDLGLE	Large Dependence Low Gray Level Emphasis
LGLRE	Low Gray Level Run Emphasis
LT	Lung tumour
LTR	Long terminal repeat
MAD	Mean Absolute Deviation
MaxP	Maximum Probability
Min	Minimum
ngtdm	Neighbouring Gray Tone Difference Matrix
NSCLC	Non-small cell lung carcinoma
NTF	Nascent tumour field
NTmF	Nascent tumour margin field
ODT	Other dense tissue
OPA	Ovine pulmonary adenocarcinoma
PrT-1	Pre-tumour-1 month
PTV	Post-tumour volume
RF	Radiomic feature
RMS	Root Mean Squared
Rnge	Range
RobMAD	Robust Mean Absolute Deviation
RunEntr	Run Entropy
RunLNUnif	Run Length Non-Uniformity
SizeZNUnif	Size Zone Non-Uniformity
SizeZNUnifNorm	Size Zone Non-Uniformity Normalized
Skew	Skewness
SmDE	Small Dependence Emphasis
SmDHGLE	Small Dependence High Gray Level Emphasis
TLV	Total lung volume
TmF	Tumour margin field
ZoneEntr	Zone Entropy
90PCNT	90th Percentile
