# Supplementary figures and images for: Radiomic Feature Characteristics of Ovine Pulmonary Adenocarcinoma
Source: Vet Sci. 2025 Apr 23;12(5):400. doi: 10.3390/vetsci12050400 (PMC12115574; doi:10.3390/vetsci12050400)

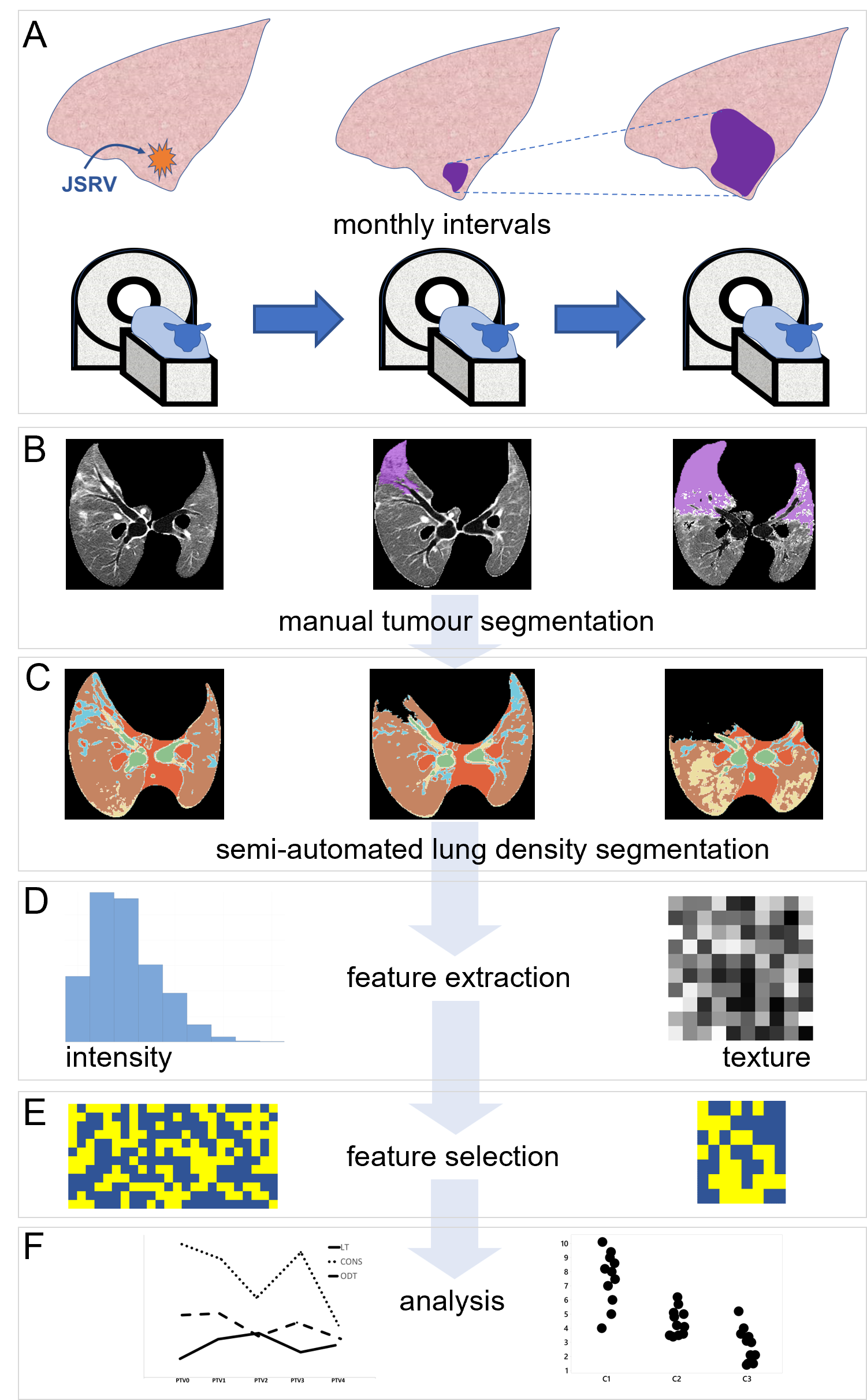

Supplement: Supplementary file 1 [file vetsci-12-00400-s001.zip › vetsci-3544338-supplementary.tif]
